# Digital Media and Developing Brains: Concerns and Opportunities

**DOI:** 10.1007/s40429-024-00545-3

**Published:** 2024-03-04

**Authors:** John S. Hutton, Jessica Taylor Piotrowski, Kara Bagot, Fran Blumberg, Turhan Canli, Jason Chein, Dimitri A. Christakis, Jordan Grafman, James A. Griffin, Tom Hummer, Daria J. Kuss, Matthew Lerner, Stuart Marcovitch, Martin P. Paulus, Greg Perlman, Rachel Romeo, Moriah E. Thomason, Ofir Turel, Aviv Weinstein, Gregory West, Pamela Hurst-Della Pietra, Marc N. Potenza

**Affiliations:** 1 Division of General and Community Pediatrics, University of Cincinnati College of Medicine and Cincinnati Children’s Hospital Medical Center, Cincinnati, OH, USA and Division of General and Community Pediatrics, University of Texas Southwestern Medical Center, Dallas, TX USA; 2 Amsterdam School of Communication Research ASCoR, University of Amsterdam, Amsterdam, Netherlands; 3 Departments of Psychiatry & Pediatrics, Addiction Institute, Icahn School of Medicine at Mount Sinai, New York, NY USA; 4 Division of Psychological and Educational Services, Fordham University, New York, NY USA; 5 Departments of Psychology and Psychiatry, Stony Brook University, Stony Brook, NY USA; 6 Department of Psychology & Neuroscience, Temple University, Philadelphia, PA USA; 7 Center for Child Health Behaviour and Development, Seattle Children’s Research Institute, Departments of Pediatrics, Psychiatry, and Health Services, University of Washington, Seattle, WA USA; 8 The Shirley Ryan AbilityLab & Department of Physical Medicine and Rehabilitation, Feinberg School of Medicine, Northwestern University, Chicago, IL USA; 9 The National Institutes of Health, Eunice Kennedy Shriver National Institute of Child Health and Human Development, Bethesda, USA; 10 Department of Psychiatry, Indiana University School of Medicine, Indianapolis, IN USA; 11 International Gaming Research Unit and Cyberpsychology Group, NTU Psychology, Nottingham Trent University, Nottingham, UK; 12 Departments of Psychology, Psychiatry & Pediatrics, Stony Brook University, Stony Brook, NY, USA and AJ Drexel Autism Institute, Drexel University, Philadelphia, PA USA; 13 Department Of Psychology, University of North Carolina Greensboro, Greensboro, NC USA; 14 Laureate Institute for Brain Research, Tulsa, OK USA; 15 Department of Psychiatry and Behavioral Health, Renaissance School of Medicine at Stony, Brook University, Stony Brook, NY USA; 16 Departments of Human Development & Quantitative Methodology, Hearing & Speech Sciences, and Neuroscience & Cognitive Sciences, University of Maryland College Park, College Park, MD USA; 17 Departments of Child and Adolescent Psychiatry and Population Health, New York University, New York University Grossman School of Medicine, New York, NY USA; 18 College of Business and Economics, California State University, Fullerton, CA USA; 19 Faculty of Engineering and Information Technology, The University of Melbourne, Melbourne, Australia; 20 The Isadore and Ruth Kastin Chair for Brain Research, Department of Psychology and Behavioral Science, Ariel University, Ariel, Israel; 21 Department of Psychology, University of Montreal, Montreal, Canada; 22 Children and Screens: Institute of Digital Media and Child Development, Jericho, NY USA; 23 Department of Family, Population and Preventive Medicine, Stony Brook Medicine, Stony Brook, NY USA; 24 Departments of Psychiatry, Child Study and Neuroscience, Connecticut Mental Health Center, Yale School of Medicine, Wu Tsai Institute, Yale University, New Haven, CT 06517 USA

**Keywords:** Internet addiction, Screen time, Video games, Social media, Pornography, Youth

## Abstract

**Purpose of Review:**

The incorporation of digital technologies and their use in youth’s everyday lives has been increasing rapidly over the past several decades with possible impacts on youth development and mental health. This narrative review aimed to consider how the use of digital technologies may be influencing brain development underlying adaptive and maladaptive screen-related behaviors.

**Recent Findings:**

To explore and provide direction for further scientific inquiry, an international group of experts considered what is known, important gaps in knowledge, and how a research agenda might be pursued regarding relationships between screen media activity and neurodevelopment from infancy through childhood and adolescence. While an understanding of brain-behavior relationships involving screen media activity has been emerging, significant gaps exist that have important implications for the health of developing youth.

**Summary:**

Specific considerations regarding brain-behavior relationships involving screen media activity exist for infancy, toddlerhood, and early childhood; middle childhood; and adolescence. Transdiagnostic frameworks may provide a foundation for guiding future research efforts. Translating knowledge gained into better interventions and policy to promote healthy development is important in a rapidly changing digital technology environment.

## Introduction

Digital technologies are increasingly becoming integrated into people’s lives from early stages. Given the importance of understanding how digital technologies and their uses may influence developing brains, the non-profit group Children and Screens convened a 2-day retreat in early 2020, during which a diverse, international, interdisciplinary group of academics (with respect to age, career stage, gender/sex, race/ethnicity, geographic locations (North America, Europe, Middle East), and areas of training/expertise (Psychiatry, Psychology, Neuroscience, Child Study, Pediatrics and other disciplines)) considered how types and patterns of media use may effect neurodevelopment.

The workshop divided participants into three groups based on developmental stages: [1•] early childhood including infancy and toddlerhood, [2] mid-childhood/pre-pubertal period, and [3] adolescence to young/emerging adulthood. The groups met partially in parallel with cross-group interactions to discuss existing data of media’s impacts on neurodevelopment and brain function from molecular, cellular, circuit-based, and systems perspectives and how these may relate to cognition and behavior through development. One framework for understanding how types and patterns of digital technology use or screen media activity (SMA) may relate to neurodevelopment involves the research domain criteria (RDoC), and this was selected prior to the meeting in order to structure and facilitate discussions regarding brain-behavior relationships [1•]. The RDoC framework is comprised of six higher-order systems [1•]: Negative Valence, Positive Valence, Cognitive, Social Processes, Arousal and Regulatory Processes, and Sensorimotor. Each of these higher-order systems is undergirded by more focal constructs and subconstructs (e.g., the construct Reward Responsiveness is subsumed by Positive Valence) that may be sensitive to types and patterns of digital technology use across development. A developmentally informed understanding of the relations between types and patterns of specific forms of digital technology use and RDoC systems at genetic, molecular, cellular, circuitry, physiological, and behavioral levels may complement other frameworks that, for example, focus on environmental contexts and guide prevention, treatment, policy, and public health initiatives, particularly given the public’s interest in brain mechanisms underlying healthy and maladaptive behaviors. The following summarizes and extends the conference work.

## Early Childhood: Digital Media, Brain Development, and Parent–Child Interactions

The span of rapid brain growth during early childhood is recognized as foundational for cognitive, relational, and social-emotional development [2]. The brain doubles in size in the first year of life and increases to about 80% of adult size by age 2, when essential structural and functional frameworks are established [2]. Brain development is shaped via network reorganization and remodeling in response to genetic and environmental factors [2]. Networks for basic skills such as vision and hearing mature early and rapidly, while those for more complex skills such as language and executive function mature gradually, yet have peak trajectories before age 5 [2]. Learned skills such as reading may rely on integration of these networks.

Effects of digital media may begin when brain networks are developing quickly and plasticity is high. Additionally, sex differences may influence susceptibilities, notably for networks involved with visual processing, language, and social cognition [3]. Disparities in constructive and/or adverse experiences linked to socioeconomic status (SES) [4] and temperament [5] also likely fuel differential effects. Complex eco-biological milieus highlight differential susceptibilities where SMA may influence neurodevelopment in non-uniform ways [6, 7].

As media exposure is largely controlled and modeled by parents and other caregivers during early childhood, caregiver perceptions and habits are important. While reasons for child media use may range from entertainment to respite from stresses of childcare, a frequent motivator may involve fueling learning and creativity, terms often used in marketing of digital content [8]. However, parental media use can affect parent–child dynamics by generating distractions from children (“technoference”) [9]. In addition to modeling excessive use, inopportune parental media use on dyadic engagement may interfere with optimal bonding and attachment.

Digital media use may exert direct or indirect effects on children’s skills, relationships, and developing brains [10]. Direct effects involve interactions with and influences arising from media content, and these may influence behavioral or perceptual functioning (e.g., interfering with sleep). Indirect effects refer to impacts that digital media can have on family and community dynamics and interactions (e.g., early attachment). As parents are described as a child’s “first and most important teachers,” indirect effects may be particularly impactful at early ages. While benefits are possible, as in co-viewing favorite shows and/or using these as inspiration for creative play, most research involving young children cites cognitive and other health risks [11]. These may be at least in part attributable to constraints on neural development and learning that have evolved in terms of multi-sensory (analog) stimuli and social contexts [12].

Few studies have directly explored relationships between digital media use and structural brain development during early childhood. Higher use defined in American Academy of Pediatrics (AAP) guidelines was associated with lower microstructural integrity of white-matter tracts supporting language and literacy in preschool-age (3- to 5-year-old) children and with lower language and literacy skills [13•]. Suggested mechanisms included displaced nurturing experiences such as parent–child (“shared”) reading which has been associated with higher microstructural integrity in tracts supporting language and literacy and higher skills in these domains [14]. In preschool-age children, higher media use has been associated with thinner cortical gray matter and shallower sulcal depth in areas involved in visual processing, social cognition, attention, and emergent literacy skills [15], consistent with findings in 10-year-old children [16•].

Two functional magnetic resonance imaging studies have described differences of within- and between-network functional connectivity during stories presented to preschool-age children in three formats: audio (listening with no pictures), illustrated (similar to a typical picture book), and animated [17, 18]. The illustrated story was associated with stronger connectivity between language, visual, cerebellar, and default-mode networks, suggesting that illustrations and imagery support age-appropriate scaffolding of language networks to support comprehension [18]. By contrast, during animated stories, connectivity between these networks was reduced [14], while maximal connectivity occurred between the dorsal-attention and visual-perception networks, suggesting hyper-engagement of the visual-perception network at the expense of network integration [18]. During animated stories, connectivity between ventral attention and language and imagery networks was lower, suggesting less dynamic reorienting due to strained working-memory capacity, consistent with behavioral evidence of animated programming reducing executive-function skills (i.e., refocusing on task) in preschool-age children [18]. This is also commensurate with evidence from a pilot intervention study in which preschool-age children exposed to 6 weeks of dialogic reading performed better on language and executive-function assessments and attention/inhibition tasks, compared to children who were presented the same stories via video [19].

There is conflicting evidence regarding associations between digital media use and child language, although there is more consensus that use at younger ages may confer greater risk. Differences in early language development through human versus screen-based instruction have been reported [20]. Using eye-tracking and electroencephalography, researchers found that infants learn phonemes via human presentations, but not television or audio recordings [21]. A suggested mechanism is that human interaction, including caregiver faces and child-directed speech, socially “gates” learning of speech cues [20]. Another catalyst may involve joint attention (i.e., parent–child-object), which begins to emerge at 5 months and involves prefrontal, visual, limbic, and other brain regions. This is exemplified by shared book reading or play, where an adult provides critical scaffolding and encouragement for the child that is not authentically reproducible by a digital device. Beyond phonetic mastery, rapid vocabulary expansion and syntax acquisition coincide with the organization and myelination of white-matter tracts, such as the arcuate fasciculus, which are sensitive to “serve and return” practice [22]. Plasticity supporting each of these abilities peaks in the first 3 years [23], underscoring the importance of nurturing dyadic language experiences in early childhood.

How technology is used contributes importantly to outcomes. For example, for young children, video-based platforms may influence how efficiently and effectively skills are acquired (referred to as a “video deficit”) [24]. There are also industry efforts focused on enhanced learning technologies. A critical factor may involve the involvement of a mature facilitator, such as an older sibling, parent, or teacher, given that early learning has evolved in social contexts centered around contingent, caregiver-child interactions [25–27]. Thus, irrespective of platform, screen-based learning may be optimized via scaffolding provided by engaged caregivers or peers [28].

Mechanistically, brain-to-brain neural synchrony has been described in preschool-age children as a biomarker of interaction quality and emotional regulation (e.g., calming) [29, 30]. Behaviorally, such synchrony is exemplified by child-directed speech and dialogic reading. Genuinely shared, nurturing experiences may activate physiological oscillator systems, such as biological clocks, cardiac pacemakers, and attachment hormones like oxytocin [31]. A key question is to what degree digital media can recreate and/or foster reciprocal human dynamics that trigger hardwired physiological and neurobiological synchronies. This is the rationale behind exemption of video chat from AAP screen-time recommendations, framed as a reasonable proxy [11]. For toddlers (12–30 months), evidence is inconsistent on learning through interactive video platforms, ranging from some learning in socially contingent “video chat” to negligible learning in scenarios with less scaffolding [32–34]. Additionally, certain features of digital media have been identified as problematic, notably distracting “enhanced” content in e-books, which may impair dyadic engagement (and perhaps neural synchrony) and comprehension [35]. However, for typically developing preschool-age children, some “learning” apps may have benefits, particularly for early math skills [28], perhaps due to less reliance on social contexts or more rote learning processes. Likewise, some digital media (e.g., augmentative/alternative communication devices) may offer benefits in terms of social accommodations and learning for children with autism spectrum disorders [36]. An unanswered question involves the abilities of children to transfer on-screen skills to the real world, which tends to be low even in adults [37].

Social cognition is a critical developmental domain supported by the default-mode network (DMN), a higher-order functional network that undergoes prolonged maturation beginning in infancy and is largely involved with self-oriented processes (e.g., theory of mind, empathy), episodic memory, and scene construction (e.g., imagery, creativity) [38]. DMN subsystems are anti-correlated with “active-task” networks, particularly those involving concentrated visual stimuli such as video viewing, even in infancy [39]. A video-deficit effect in social cognition has been described in preschool-age children, in which abilities to perceive others’ mental states were lower during video compared to real-world situations [40], which may in part be fueled by screen-inhibitory effects on DMN engagement. Speculatively, excessive SMA during early childhood may blunt social cognition in certain subgroups of children via DMN inhibition [41].

Considerable research is needed to address major unanswered questions regarding effects of digital media use on developing brains across early childhood, ideally beginning prenatally. The major themes suggested are summarized in Table 1. Central to the themes are neurobiological readiness, roles of human caregivers (dyadic relationships), differential susceptibilities across youth, and what may be expected in terms of learning and health from media use. A multi-tiered, longitudinal approach using multiple tools, including neuroimaging, hormonal and (epi-)genetic assays, validated cognitive/behavioral assessments and surveys, and reliable measures of digital media use, has the potential to provide critical insights for typically developing children and characterizing those most at-risk. Meanwhile, it is important to view digital media appropriately as powerful tools akin to fire or automobiles, where caution is warranted and reasonable age restrictions apply, particularly when children are most vulnerable.

**Table 1:** Topics suggested for further research

Topic	Subtopics—early childhood	Subtopics—middle childhood	Subtopics—adolescence
Relationships/neural and physiological synchronies	Dyadic relationships, child use, parent use, simultaneous use (co-viewing vs parallel), effects on learning and relationships (features, content, context), technoference	Child and tween use, parent use, simultaneous use, effects on learning and relationships (features, content, context), media multitasking	Teen use, parent use, simultaneous use, effects on learning and relationships (features, content, context), media multitasking, peer relationships/social use
Evolutionary constraints	Social context, multi-sensory processing, learning through play, reliable contingency	Metamemory, selective attention, spatial and mathematical reasoning, reading fluency and comprehension, argumentation, theory of mind, and executive function including working memory and flexibility	Pubertal hormones impact biological systems and may contribute to emotional dysregulation and engagement in risky behaviors. Subcortical brain regions and networks that may underlie strong emotions or promote impulsive behaviors typically mature more rapidly than prefrontal cortical ones that exert control over emotions and behaviors
Cognitive skills	Social cognition (basic attachment, empathy), language, executive functions (self-regulation, response, inhibition, distress tolerance), attention (top-down focus, bottom-up flexible reorienting), higher-order visual/imagery, emergent literacy/reading	Theory of mind, metacognition, executive function, visual processing, impulsivity, academic performance	Theory of mind, metacognition, executive function, visual processing, impulsivity, academic performance
Differential susceptibilities	Age, gender, temperament, family history (addiction, mood disorders, high vs. low media use), SES	Age, gender, temperament, family history (addiction, mood disorders, high vs. low media use), SES, psychopathologies	Age, gender, temperament, family history (addiction, mood disorders, high vs. low media use), SES, psychopathologies
Using phenotyping	Risk categories (e.g., mood, addictive, aggressive, OCD)	Risk categories (e.g., mood, addictive, aggressive, OCD)	Risk categories (e.g., mood, addictive, aggressive, OCD)
Use during school and in formal settings	Learning potential, domains more/less compatible with digital platforms (language, literacy, creativity, physical education, social skills, math), effect of media content and features (interactive, pacing)	Learning potential, domains more/less compatible with digital platforms (language, literacy, creativity, physical education, social skills, math), effect of media content and features (interactive, pacing)	Learning potential, domains more/less compatible with digital platforms (language, literacy, creativity, physical education, social skills, math), effect of media content and features (interactive, pacing)
Neuroimaging	Structural correlates (MRI—thickness, gyri, DTI), functional correlates (EEG, fNIRS, fMRI RS/task, eye tracking), neurotransmitters (dopamine, GABA, glutamate), naturalistic vs lab, awake vs asleep	Structural correlates (MRI—thickness, gyri, DTI), functional correlates (EEG, fNIRS, fMRI RS/task, eye tracking), neurotransmitter (dopamine, GABA, glutamate), naturalistic vs lab, awake vs asleep	Structural correlates (MRI—thickness, gyri, DTI), functional correlates (EEG, fNIRS, fMRI RS/task, eye tracking), neurotransmitter (dopamine, GABA, glutamate), naturalistic vs lab, awake vs asleep
Biomarkers	Neurotransmitters (dopamine, serotonin), hormones (cortisol, melatonin, oxytocin), genetics and epigenetics (methylation)	Neurotransmitters (dopamine, serotonin), hormones (cortisol, melatonin, oxytocin), genetics and epigenetics (methylation)	Neurotransmitters (dopamine, serotonin), hormones (cortisol, melatonin, oxytocin), genetics and epigenetics (methylation, histone modifications, chromatin remodelling)
Reliable, multi-faceted screening of parent and child	Parent report surveys (ScreenQ composite), objective measures (recorders, embedded apps)	Objective measures (recorders, embedded apps), school guidance counselor reports, academic reports, parent report surveys	Objective measures (recorders, embedded apps), school guidance counselor reports, academic reports, parent report surveys
Effect of interventions	Prenatal, infant/toddler, preschool, by setting	School-based, home-based, care-based, for different elements of digital media use, for addiction, for problematic social media use	School-based, home-based, care-based, for different elements of digital media use, for addiction, for problematic social media use
Differential effects of differential use on brain structure and function	Time, content, context, character of the child	Video gaming, social media, pornography, creative apps/opportunities, cyberbullying, smartphone use, media multitasking; time, content, context	Video gaming, social media, pornography, creative apps/opportunities, cyberbullying, smartphone use, media multitasking; time, content, context
COVID-19	How pandemic screen use has impacted typical brain development	How pandemic screen use has impacted typical brain development	How pandemic screen use has impacted typical brain development

Abbreviations: *DTI* diffusion tensor imaging, *EEG* electroencephalogram, *fMRI* functional magnetic resonance imaging, *fNIRS* functional near-infrared spectroscopy, *GABA* Gamma-aminobutyric acid, *MRI* magnetic resonance imaging, *OCD* obsessive–compulsive disorder, *RS* resting-state, *SES* socioeconomic status

## Middle Childhood: Emerging Individuation

Middle childhood, from approximately 6 to 12 years of age, is an often-forgotten period for studying media effects on behavior. Children in middle childhood are less frequently studied than their older and younger peers. This period, however, is arguably as important as others.

Cognitively, this period is marked by enhancements that influence how children engage in the world. Changes include increasing sophistication in metamemory, strategies to recall content, selective attention, spatial and mathematical reasoning, reading fluency and comprehension, argumentation, theory of mind, and executive function, including working memory and flexibility [42]. During this period, psychopathology often begins, with nearly 50% of all mental illnesses diagnosed before adolescence [43].

Social-emotional development and corresponding desires for autonomy progress during middle childhood. Advancing cognitive functioning leads to improved understandings of others’ perspectives and emotions, which in turn facilitate building and maintaining peer relationships.

With cognitive and social-emotional changes, children’s SMA preferences and behaviors also change. During middle childhood, children often use media for leisure, with an estimated average SMA of more than 4 h/day and with higher use for children from lower-income families [44]. Consistent with desires for greater autonomy, this period often includes the acquisition of the first personal smartphones. By the age of 12, 69% of US children have their own smartphone, and this rate continues to rise as smartphones increase in accessibility [44]. Media use is also different from early childhood, with online videos and gaming consuming substantial portions of media time [44] and social media consuming less time when compared to adolescents.

Middle childhood is understudied with respect to media access, consumption, and effects when compared to early childhood and adolescent groups. While several studies examine middle childhood regarding media use in formal settings and the acquisition of content knowledge and general skills, few have investigated heterogeneity of access and processing of media content in middle childhood, particularly in informal (home) settings [42].

Neurodevelopmentally, brains reach peak sizes between 10 and 12 years, in girls typically earlier than in boys [45]. Subsequent cortical thinning generates reduced gray matter volume, in part due to synaptic pruning. Concurrently, roughly linearly increasing white matter improves neural communication [46]. Delays in neurodevelopmental trajectories are associated with executive-functioning deficits, such as those in attention-deficit/hyperactivity disorder [46].

Neurodevelopmental delays underlying poor cognitive skills may relate to the use of digital media. A bidirectional relationship between impulsivity and video gaming during childhood exists, suggesting that gaming may be both a cause and effect of increased neurodevelopmental delays, impulsivity, and attentional problems [47], although some data suggest positive cognitive correlates of gaming [48, 49]. Among 4277 children aged 10 years from the ongoing nationwide Adolescent Brain and Cognitive Development (ABCD) study, a negative correlation between SMA and cortical thickness was observed [16•]. These findings suggest that higher SMA may relate to slower cortical maturation. The study also associated SMA with crystalized and fluid intelligence and observed a positive association between SMA and externalizing but not internalizing psychopathology. This work aligns with studies from the same cohort associating higher SMA with lower global cognition, higher impulsivity, and binge-eating behaviors [50]. Relationships may extend to academic performance, with more television use among 8–9-year-old children associated with decreased literacy 2 years later [51]. The extent to which these relationships are driven by SMA or decreased time with displaced activities (sleep, reading) is unclear, particularly as brain connectivity between visual processing and higher-level-cognition/language regions has been associated positively with reading time and negatively with SMA during middle childhood [52].

More recently, a cross-validated latent profile analysis of ABCD baseline (9–10-year-old) youth identified seven empirically derived, brain-based developmental subgroups, with six of the groups characterized by high and low reward responsiveness, inhibition, and emotional regulation and most (two-thirds) being a moderate group. Relative to the moderate group, the other six groups were characterized by being male and from lower-income households and having poorer cognitive performance, more SMA, heightened impulsivity, and more neurodevelopmental disorders [53•]. Independently, a brain structural covariation pattern linked to underage alcohol consumption in adults was replicated (*r* = 0.99) in ABCD baseline participants and linked to SMA and externalizing behaviors [54•]. Further, changes in a similar brain structural covariation pattern mediated the relationship between SMA at the ABCD baseline and internalizing concerns 2 years later [55•]. A videocentric high-SMA pattern identified in 26.7% of the baseline ABCD sample increased to 30.4% at 1-year follow-up [56•]. Importantly, the high-SMA group at baseline exhibited poorer neurocognitive performance, more behavioral problems, more severe prodromal psychotic concerns, higher impulsivity, and more sensitivity to punishment/reward and altered resting-state functional connectivity among brain areas implicated previously in cognitive processes, with relationships persisting over the following 2 years [56•]. Replication of the videocentric high-SMA pattern was observed in a separate dataset, with youth from the latter exhibiting a socio-communication-centric high-SMA pattern from age 13 on [56•].

During middle childhood, relationships between media use, cognitive and social-emotional development, and psychopathology may be unfolding. There are many less well-studied aspects, including brain-behavior relationships linked to specific types and patterns of digital technology use that are emerging. In such studies, transdiagnostic and clinically relevant measures should be considered, as this period holds significant opportunities for interventions to be developed.

## Adolescence: Individuating into Adulthood

Adolescence is the period of transition from childhood to adulthood. Precise definitions of the beginning and end of adolescence vary, with the World Health Organization defining the period from 10 to 19 years of age [57] and the AAP defining it from 11 to 21 years [58]. Adolescence has also been considered commencing with puberty and ending with becoming legally of majority age, which is 18 years in the USA, although some behaviors (e.g., alcohol consumption) are not legally permitted until age 21. Given definitions of adolescence spanning from puberty to the onset of adulthood, the age range may vary based on individual developmental and cultural factors.

During adolescence, pubertal hormones impact biological systems and may contribute to emotional dysregulation and engagement in risky behaviors. Subcortical brain regions and networks that may underlie strong emotions or promote impulsive behaviors typically mature more rapidly than prefrontal cortical ones that exert control over emotions and behaviors, leading to adolescence being a period of addiction vulnerability [59]. Adolescence is marked by onset and high rates of addictive behaviors, including substance use and gambling disorders [60]. Digital technologies offer additional outlets for possible addictive engagement as they may deliver intermittent rewards (e.g., social media “likes,” loot box contents) that may promote continued use. Digital technologies also offer many benefits for adolescents, depending on types and patterns of usage, with moderate use typically associated with better health, including less substance use. However, excessive use may negatively influence multiple domains of functioning, either directly or indirectly, including through sleep impairment, poor social cognition, or worsening executive functioning. As heavier media multitasking has been linked to poorer memory, increased impulsivity, and decreased volume in brain regions linked to cognitive control and emotional regulation [61], even SMA often considered “non-problematic” may have negative impacts on developing brains with effects on real-world functioning like driving safety.

Types and patterns of media use vary considerably among adolescents. Most adolescent use of digital technologies is conducted on mobile devices, with the vast majority of US teenagers reporting access to smartphones and platforms such as YouTube, TikTok, Snapchat, and Instagram being most popular, used by 93%, 63%, 60%, and 59%, respectively [62]. Patterns of social media use are important. A longitudinal study of 6595 adolescents found that spending more than 3 h/day on social media was associated with an increased risk for mental health problems, particularly depression [63], consistent with a recent US Surgeon’s General report [64]. The popularity of social media speaks to the importance of social interactions among adolescents, highlighting the need for more research into possible positive and negative health correlates.

Online gaming is popular among adolescents and may have positive and negative effects. Gaming promotes social inclusion among adolescents, particularly boys [65]. Gaming may have cognitive benefits [48], and further investigations of brain mechanisms are warranted. However, excessive patterns of gaming may generate clinical concerns, and thus research criteria have been included in DSM-5 for internet gaming disorder (IGD) [66], and gaming disorder (GD) has been included in the ICD-11 [67]. Although adolescent boys seem particularly vulnerable to developing problems with gaming, gaming may constitute a hidden problem in adolescent girls [68]. Motivations for gaming are important to consider, as escape and coping motives (negative reinforcement motivations) have been linked to IGD [69]. Other mental health concerns (e.g., depression, attention-deficit/hyperactivity disorder) often co-occur with IGD, and initial studies are providing insight into underlying processes and brain mechanisms [70].

Given both positive and negative impacts of gaming, the World Health Organization and other groups have advocated for balanced engagement in gaming and other forms of internet use, especially during the COVID-19 pandemic [71]. The pandemic may influence multiple types and patterns of internet use for adolescents (remote schoolwork, searching for health information potentially leading to cyberchondria [71]), and understanding its influences on adolescent development will be important. How best to define non-gaming problematic forms of internet use has been debated, with one option being as “other specified disorders due to addictive behaviors” once ICD-11 is implemented in jurisdictions [72•].

Given clinical implications, arguably more neurobiological research has been conducted on problematic patterns of internet use, particularly gaming in adolescent and young adult males. Both empirical and theoretical neurobiological support exists for IGD’s/GD’s classification as addictive disorders [73]. While similar considerations appear to hold for other patterns of excessive internet use [74•], debate remains regarding their best classification. A meta-analysis indicated smaller brain volumes in individuals with IGD, including in frontal cortical areas implicated in motivation and cognitive control [73]. The meta-analysis also found between-group differences (IGD vs non-IGD) in cortical and subcortical brain function during “hot” and “cold” executive processes. While structural and functional brain features have been linked to other forms of problematic/addictive internet use (e.g., social network use [75], problematic pornography use [76], and internet addiction [77]) and brain-behavior models have been proposed involving specific cortical and subcortical brain circuits (e.g., fronto-striatal circuitry involving the dopaminergically innervated striatum) [74•], such models should be placed into neurodevelopmental contexts. More research, particularly longitudinal, is needed to understand how specific types and patterns of internet use may impact adolescent neurodevelopment and behavior.

## Conclusions

The Digital Media and the Developing Brain Retreat facilitated sharing important insights and identifying areas vitally needing further study. A cohesive, collaborative research agenda spanning developmental epochs can help understand how types and patterns of digital technology use may influence brain structure and function and cognition and behavior. This research should consider the multiple RDoC units and domains with a focus on improving the lives of children and families (Table 1). Additional frameworks should also be considered, incorporating neurodevelopmental perspectives. For example, differential susceptibility considers genetic and environmental risk factors fueling maladaptive use that may be identified and addressed early. An online-offline integration hypothesis has been proposed with initial support in adults [78, 79], and this work should be extended to understand brain-behavior relationships in youth. Other models proposing frameworks for problematic use of the internet (e.g., the Interaction of Person, Affect, Cognition, and Execution) also provide meaningful structures for understanding brain-behavior relationships and how individual differences may operate as vulnerability or resilience factors throughout time [74•, 80]. Of note, the I-PACE model considers SMA within an addiction framework, consistent with data suggesting that specific types and patterns of internet use may constitute disorders due to addictive behaviors [72•]. Considering specific types and patterns of SMA within an addiction framework may guide regulatory efforts. Additional conceptual frameworks (e.g., involving a Bronfenbrenner model) have incorporated multi-level considerations for how SMA may relate to mental health [81], in line with the recent US Surgeon General’s guidance on social media use and youth mental health [82]. Additional longitudinal studies are warranted to examine the validity of these proposed frameworks and to identify related brain mechanisms and their potential clinical utility.

Practically, brain measures are currently not typically incorporated into clinical settings when considering effects of SMA, given high costs and current lack of clinical guidelines based on potential findings. While such information may ultimately help as one progresses toward personalized interventions, currently more refined behavioral assessments (e.g., through direct measurement of SMA rather than parental or self-report) and deep phenotyping of contextual factors (e.g., school, home, and peer environments) are likely to provide important, practical information. Given that public interest exists in understanding brain mechanisms and the relevance that brain measures may have in complementing other domains, large-scale initiatives like the HEALthy Brain and Child Development Study (HBCD) and ABCD have incorporated brain imaging measures to better understand relationships between brain structure and function and measures of infancy/toddlerhood, childhood, and adolescent functioning and well-being. Important unanswered questions may be addressed as such data are collected. For example, given gender-/sex-related differences in IGD and problematic use of social media in adults [83] and apparent transitions from video-centric to socio-communication-centric patterns of SMA around age 13 [56•], will different types and patterns of excessive SMA affect different groups of youth and relate to different brain mechanisms and outcomes? Such information may be used to update SMA guidelines from the AAP and other organizations, as well as to inform resources for parents and other interested stakeholders [84•].

Many questions currently remain unanswered or incompletely understood. Longitudinal research investigating bi-directional relationships between SMA and youth outcomes should provide greater insight, with brain measures offering insight into possible mechanisms and in the case of excessive SMA, potential therapeutic targets (85). Multiple barriers exist (need for large studies with opportunities for replication to ensure rigor and reproducibility, limitations of current analytic approaches, often small magnitudes of specific brain effects, challenges of imaging youth with respect to movement, to name several). In this process, multiple imaging approaches and analytic methods and incorporation of deeply phenotyped samples with respect to types and patterns of SMA, environmental factors, and other individual differences are needed longitudinally throughout development. Some imaging approaches may be less susceptible to movement, less intimidating to youth, and more feasible for collecting data on relevant behaviors within specific environmental contexts (e.g., in family or dyadic settings), and these should be considered for investigating both potentially beneficial and detrimental aspects of SMA on development. Through such research, targeted interventions (policy, prevention, treatment) may be developed to help promote healthy cognitive and social-emotional development from infancy through adulthood.

## Abbreviations

AAP: American Academy of Pediatrics
ABCD: Adolescent Brain Cognitive Development Study
DSM-5: Fifth edition of the Diagnostic and Statistical Manual
DMN: Default-mode network
GD: Gaming disorder
HBCD: HEALthy Brain and Child Development Study
ICD-11: Eleventh revision of the International Classification of Diseases
IGD: Internet gaming disorder
RDoC: Research domain criteria
SES: Socio-economic status
SMA: Screen media activity
